# Validity evidence and reliability of a simulated patient feedback instrument

**DOI:** 10.1186/1472-6920-12-6

**Published:** 2012-01-27

**Authors:** Claudia Schlegel, Ulrich Woermann, Jan-Joost Rethans, Cees van der Vleuten

**Affiliations:** 1 Skillslab, Berner Bildungszentrum Pflege, Reichenbachstrasse 118, 3004 Berne, Switzerland; 2 Institute of Medical Education, Education and Media Unit, Medical Media Production, University of Bern, Berne, Konsumstrasse 13, 3010 Berne, Switzerland; 3 Skillslab, Faculty of Health, Medicine & Life Sciences, Maastricht University, Netherlands, PO Box 616, 6200 MD Maastricht, The Netherlands; 4 Department of Educational Development and Research, University of Maastricht, Netherlands, P.O. Box 616, University of Maastricht, 6200 MD Maastricht, The Netherlands

## Abstract

**Background:**

In the training of healthcare professionals, one of the advantages of communication training with simulated patients (SPs) is the SP's ability to provide direct feedback to students after a simulated clinical encounter. The quality of SP feedback must be monitored, especially because it is well known that feedback can have a profound effect on student performance. Due to the current lack of valid and reliable instruments to assess the quality of SP feedback, our study examined the validity and reliability of one potential instrument, the 'modified Quality of Simulated Patient Feedback Form' (mQSF).

**Methods:**

Content validity of the mQSF was assessed by inviting experts in the area of simulated clinical encounters to rate the importance of the mQSF items. Moreover, generalizability theory was used to examine the reliability of the mQSF. Our data came from videotapes of clinical encounters between six simulated patients and six students and the ensuing feedback from the SPs to the students. Ten faculty members judged the SP feedback according to the items on the mQSF. Three weeks later, this procedure was repeated with the same faculty members and recordings.

**Results:**

All but two items of the mQSF received importance ratings of > 2.5 on a four-point rating scale. A generalizability coefficient of 0.77 was established with two judges observing one encounter.

**Conclusions:**

The findings for content validity and reliability with two judges suggest that the mQSF is a valid and reliable instrument to assess the quality of feedback provided by simulated patients.

## Background

A major advantage of communication training with simulated patients (SPs) in the training of healthcare professionals is that SPs are able to provide feedback to students from a patient's perspective immediately after a simulated clinical encounter (SCE) [1-3]. Feedback is an important and valuable tool in interactive learning [4], and for our purposes, it may be defined as the provision of specific information on a student's performance relative to a specific performance standard [5], with the intention of improving the student's performance. High-quality feedback can have a profound effect on student performance [6] and is, therefore, vital to the overall effectiveness of a training sequence.

To assess the quality of SP feedback, a valid and reliable assessment instrument is needed. The only existing instrument, the "Maastricht Assessment of Simulated Patients" (MaSP) [7], has two subscales and assesses both the quality of SP feedback and the authenticity of SP performance during a simulated consultation; as a result, it is not detailed enough to assess the quality of SP feedback in-depth. At the same time, some items of the MaSP, e.g. "SP left the room between consultation and feedback", are too specific and irrelevant for institutions where the SP stays in the room between encounter and feedback.

In the "grey literature", we found another instrument, the "Quality of Simulated Patient Feedback (QSF) Form" [8], which was designed to help SP trainers to evaluate the quality of the oral SP feedback process and thus to determine whether SPs need more training in oral feedback. The detailed items of this instrument meet our needs, but the form has never been validated and there are no data on its reliability. The QSF is an 18-item questionnaire (Table 1) with a dichotomous checklist based on the tenets of basic feedback rules [9-11]. For our purpose, the dichotomous rating options were expanded to a four-point rating scale (c.f. the method section for reasons). The aim of our study, then, was to gather evidence on the validity and reliability of the mQSF when used to assess the quality of feedback provided by SPs.

## Methods

The study was conducted at a school of nursing in Berne, Switzerland, with nursing students in their second of three curricular years. The two-step approach of the study consisted, first, of an evaluation of the evidence for content validity, and second, of a generalizability analysis to estimate the reliability of the instrument.

### Forward-backward translation of the questionnaire

Since the study was conducted in a German-speaking country, the English QSF had to be translated into German. We used a forward-backward translation approach, which is recommended for translating test instruments [12]. Using this approach, a native speaker of the target language (in our case German) translated the instrument from the source language (English), and another person fluent in English then translated the text back from German into English. The original and the back-translated versions were then compared to ensure that the meaning and the nuances of the text were conserved.

### Evidence for the content validity of the mQSF items

The content validity of the 18 mQSF items was ascertained by asking 25 medical and nursing education experts from Switzerland, Germany and Austria to rank the importance of each item on a four-point rating scale (1 = not at all important; 4 = very important), using an online survey tool. An even number of scale points (no "neutral" middle position) was used to force clear ratings. The experts were alumni of the Master of Medical Education Programme at the University of Berne, Switzerland, who were actively involved in SP programmes at their own institutions. They were also invited to comment on the mQSF, e.g. whether they thought additional items should be added.

Moreover, since the items were rated on an ordinal rating scale, both mean and median ratings were calculated. Further, Cronbach's α was calculated to ascertain homogeneity among raters. An item-total correlation was performed to check whether any item is inconsistent with the rest of the scale and would thus have to be discarded.

We considered the relevance of an item of the mQSF as most important. If the mean of such an item was below 2.5 we studied the item-correlation of that item in more detail and decided to withdrew that item if a negative item-total correlation was present.

### Reliability of the mQSF

We were interested in the reliability of the quality of the SP feedback and of how the quality might be increased, e.g. by having more than one judge rating the quality. For this purpose, an analysis of generalizability (using Genova [13]) was used; reliability estimates were based on a partitioning into *true *and *multiple sources of error *variance.

Six SPs were videotaped during eight clinical encounters with different students; at the end of each encounter, feedback was given by the SPs. One videotaped encounter per SP was randomly selected for assessment by ten faculty members who judged the feedbacks according to the mQSF items. The six SPs, four females and two males, had at least 1 year of experience in role-playing and giving feedback. Three SPs impersonated a case of acute postoperative pain after an open appendectomy and were instructed to act as if they were afraid that something had gone wrong during the operation. The other three SPs enacted the role of a patient in a consultation on oral anticoagulation therapy after aortic valve replacement; they were instructed to act as if they were indifferent toward the information they received. All SP clinical encounters used and recorded in this investigation were specifically designed for this purpose and in line with the heretofore-acquired curricular competences.

In the G-study, the quality of feedback given in these six encounters was rated by 10 judges (teachers from our institution who were trained in the use of the mQSF) using a rating scale for the mQSF that ranged from 1 (= strongly agree) to 4 (= strongly disagree). We expanded the originally dichotomous rating options to a four-point rating scale because we wanted to provide more subtle parameters for the assessment of SP performance in terms of qualitative holistic judgments [14]. Three weeks later, the procedure was repeated with the same ten teachers and the same six recorded SCEs. We thus had a fully-crossed Video (encounter) by Rater by Occasion (6×10×2) design in which we treated all facets as random.

**Figure 1:**
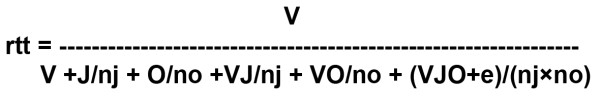
**The object of measurement for the D-study**. Facet "V" (video), of a CD-recorded clinical encounter, number (n) of judges (facet J), occasions (facet O)

In the subsequent decision-study (D-study), the facet "V" (video) of a CD-recorded clinical encounter was the object of measurement, whereas the number (n) of judges (facet J) and occasions (facet O) were varied (Figure 1).

### Ethical considerations

Ethical approval was sought from the ethics committee of the State of Bern, Switzerland. Informed consent was obtained from all participating students and SPs. Participation in the study was completely voluntary. All participants were free to leave the study at any time without any repercussions. There was no financial compensation.

## Results

### Forward-backward translation

The comparison of the original source text of the QSF and the retranslated text revealed no major discrepancies.

### Evidence of content validity of the mQSF items

Of the 25 experts invited to participate in the study, 14 completed the questionnaire (response rate 56%). The importance rates of the mQSF items from the experts were > 2.5 on a four-point rating scale for all but two items. The highest ratings were those for items 7, 8, and 10 (mean = 3.86 (SD = 0.53), median = 3.96), whereas the lowest ratings were those for items 12 (mean = 2.43 (SD = 1.28), median = 2.10) and 18 (mean = 2.43 (SD = 1.09), median 2.13) (Table 1).

**Table 1:** Experts' judgments of the importance of the QSF items.

No	Item	Mean	Standard deviation	Median	Item-total correlation
1	SP: *So, how do you think it went?*	3	1.11	3.25	0.23

2	SP: *So, what are some things you think you did well?*	3.36	1.08	3.72	0.28

3	SP: *Is there anything you would do or say differently, if you could do this again?*	3.36	0.93	3.63	0.65

4	SP gave student adequate time to answer questions before continuing	3.36	0.93	3.63	0.73

5	SP first gave positive feedback	3.14	1.13	3.63	-0.13

6	SP's positive feedback referred to specific changeable behaviours	2.93	1.14	3.00	0.70

7	SP gave feedback from patient's perspective	3.86	0.53	3.96	0.22

8	SP's negative feedback referred to specific changeable behaviours (feedback was not destructive)	3.86	0.53	3.96	0.09

9	SP limited the constructive feedback to 2 or fewer points	2.86	1.13	2.38	0.33

10	SP gave constructive feedback from patient's perspective	3.86	0.53	3.96	0.22

11	SP stopped feedback and acknowledged student's feelings	2.79	1.12	2.36	0.30

12	SP confirmed the feelings with student	2.43	1.28	2.10	0.74

13	SP reassured student about purpose of feedback	3.14	1.03	3.63	0.60

14	SP finished feedback on a positive note	2.57	0.94	2.20	0.71

15	SP asked student to summarize feedback given	2.86	1.23	3.00	0.33

16	The SP ensured that the student understood what she (the student) needed to work on	2.79	1.31	3.00	0.36

17	SP continued to ask student if she had questions until student said "no"	2.79	1.12	2.36	0.43

18	SP thanked the student	2.43	1.09	2.13	-0.18

The items were largely, albeit not completely, homogeneous for all judges, with a Cronbach's α of 0.78. As for the item-total correlation, items 5 and 18 showed negative correlations with the overall score. The latter item was also rated with a mean < 2.5, which is why we excluded it. Without item 18, the internal consistency (Cronbach's α) increased from 0.78 to 0.80. None of the experts gave comments on adding new, or modifying existing, items.

### Reliability of the mQSF

All simulated clinical encounters, including the feedback part, were conducted and recorded successfully.

The estimated variance components that resulted from the analysis of variance are given in Table 2. Most of the variance can be explained as systematic differences between videos (70%). Being the object of measurement, this represents the true score variation. The general error term is the largest source of error, followed by systematic variation between the raters (rater leniency/stringency; nearly 8%) and rater by video variation (rater leniency/stringency for some videos, but not for others; 16%). All occasion-related components are small, indicating a high intra-rater consistency.

**Table 2:** Estimated variance components

	Variance components		
**Source**	**Estimate**	**Error**	**% of total Variance**

V	79.390	43.905	70.33
J	8.714	6.279	7.72
O	0.714	1.035	0.63

VJ	18.198	5.957	16.12
VO	0.000	0.867	0.00
JO	2.069	2.276	1.83

VJO	18.363	3.788	16.27

*V *video, *J *judges, *O *occasions

Using these variance components, we got a domain-referenced dependability coefficient of 0.633 was calculated by using one judge on one occasion using the formula expressing the composition of the sources of error variance divided by their respective sample size (n) (Figure 1).

The judge-related components are rather large, which means that sampling more judges would increase reliability. On the other hand, repeated judgments would hardly result in increased reliability, given the small variance component of 0.71 for facet O (occasion).

The following estimates of the dependability coefficient were calculated running D-studies for varying numbers of judges and occasions (Table 3).

**Table 3:** Number of judges, occasions and reliability

	1 judge	2 judges	3 judges
1 occasion	0.63	0.77	0.83

2 occasions	0.68	0.81	0.86

3 occasions	0.70	0.83	0.88

## Discussion

### Evidence of content validity of the mQSF

Consistently positive expert ratings appear to support the conclusion that the mQSF has adequate content validity. Cronbach's α with 17 items was 0.80, which suggests a high degree of rater homogeneity.

Correlations among items were positive, except for items 5 and 18. However, these two items differed with regard to importance, which was rated quite high for item 5 but low for item 18 (Table 1). Item 5 ("SP gave first positive feedback") relates to an essential feedback rule (sandwich technique, [15]) which holds that starting with positive feedback creates an open mind-set in the recipient of the feedback. Item 18 ("SP thanked the student"), on the other hand, addresses a cultural peculiarity. Lauffs et al. (2008) [16] state that it is not only necessary to translate an instrument from one language into another, but also to adapt it culturally. At institutions where thanking students at the end of a feedback session is not customary, item 18 should indeed be removed from the QSF as we did.

Item 12 ("SP confirmed the feelings with student") had the lowest mean and median for importance ratings (Table 1), but showed the highest correlation with the overall score. Moreover, the importance of emphasising students' feelings has been underlined by Steinwachs (1992) [17] who stated that strong feelings of students should be addressed.

### Reliability of the mQSF

The G-study shows that increasing the number of judges observing an SP giving oral feedback increases the generalizability coefficient. A realistic design would include one judge and one occasion. In our decision study, this yielded a generalizability coefficient of 0.63. Since individual judges are likely to be subjective in their judgments and can introduce substantial error variance, it seemed advisable to use more than one judge. In fact, with two judges, the generalizability coefficient increased from 0.63 to 0.77. This indicates that two judges should observe the same encounter if possible.

## Limitations

A limitation of our study is that the original instrument was translated into another language. Translating an instrument always involves the risk that the original idea expressed in an item may not be conveyed fully and accurately. Cultural differences can also hamper accurate representation of item content. A further limitation is that only content validity was explored and not other types of validity, but we studied the content validity of this instrument because without content validity, other types of validity are meaningless.

While 14 raters seem sufficient to determine content validity [18], the generalizability study involved a rather low number of simulated clinical encounters; this was due to limited resources. However, a small sample size may be problematic with respect to representatively as it limits the generalizability to other settings and the confidence in the results of the G-study. Moreover, in our study, only one encounter, one case and one student per participating SP was rated. It would be interesting to explore case variability in SP feedback in future research.

## Conclusions

The findings for content validity and reliability with two judges suggest that the mQSF is a valid and reliable instrument to assess the quality of feedback provided by simulated patients.

We recommend that more studies be conducted, with larger samples, more cases, and more students to corroborate the findings reported here. Such studies should include more encounters and compare results obtained with the mQSF with results obtained with the feedback quality component of the MaSP or other instruments for the assessment of feedback quality.

## Competing interests

The authors declare that they have no competing interests.

## Authors' contributions

UW, JJR and CvdV supervised CS in the design, data collection and statistical analyze of the project. UW, JJR, CvdV assisted CS in interpretation of results, drafting the manuscript and critically evaluated earlier drafts of the manuscript. All authors read and approved the final manuscript.

## Pre-publication history

The pre-publication history for this paper can be accessed here:

http://www.biomedcentral.com/1472-6920/12/6/prepub
